# Psychometric properties of the Japanese version of the Kansas City Cardiomyopathy Questionnaire in Japanese patients with chronic heart failure

**DOI:** 10.1186/s12955-020-01483-0

**Published:** 2020-07-17

**Authors:** Emi Watanabe-Fujinuma, Hideki Origasa, Luke Bamber, Lothar Roessig, Tetsumi Toyoda, Yuri Haga, Chad Gwaltney, Burkert Pieske

**Affiliations:** 1 Health Economics and Outcomes Research, Market Access, Bayer Yakuhin, Ltd., Tokyo, Japan; 2 Biostatistics and Clinical Epidemiology, University of Toyama Graduate School of Medicine and Pharmaceutical Sciences, Toyama, Japan; 3 Bayer AG, Wuppertal, Germany; 4 Clinical Study Support, Inc, Nagoya, Japan; 5 Gwaltney Consulting, Westerly, RI USA; 6 Department of Internal Medicine and Cardiology, Campus Virchow Klinikum, Charité University Medicine Berlin, Berlin, Germany; 7 Department of Internal Medicine and Cardiology, German Heart Center Berlin, Berlin, Germany; 8 DZHK (German Center for Cardiovascular Research), Berlin, Germany; 9 Berlin Institute of Health (BIH), Berlin, Germany

**Keywords:** Kansas City Cardiomyopathy Questionnaire, Psychometric properties, Heart failure, Japanese, Functional status

## Abstract

**Background:**

Heart failure is a worldwide health problem that significantly affects patients’ physical function and health state. The Kansas City Cardiomyopathy Questionnaire (KCCQ) is a disease-specific patient-reported outcome measure commonly used for the assessment of health states of patients with heart failure. This study aimed to evaluate the psychometric properties of the Japanese version of the KCCQ.

**Methods:**

Using pooled data of 141 Japanese patients with chronic heart failure from three clinical trials, the Japanese version of the KCCQ was evaluated for validity and reliability, with a focus on the clinical summary score (CSS) and its component domains. For construct validity, the associations of baseline KCCQ scores with New York Heart Association (NYHA) class and the EuroQol five-dimension, three-level (EQ-5D-3L) scores at baseline were analyzed. For reliability, internal consistency was assessed using Cronbach’s α, and test–retest reliability (reproducibility) was assessed among stable patients. Responsiveness to changes in patients’ clinical status was assessed by analyzing score changes between two timepoints among patients whose health states improved.

**Results:**

Among 141 patients (mean age, 73.7 ± 10.9 years), 76.6% were NYHA class II at baseline. For CSS and its component domains (physical limitations, symptom frequency, and symptom severity), baseline scores were all significantly lower in patients with a higher NYHA class (*p* <  0.001 for all, Jonckheere-Terpstra test). The physical limitations domain and CSS showed a moderate correlation (Spearman’s *ρ* = − 0.40 to − 0.54) with three functional status-related EQ-5D dimensions (mobility, self-care, and usual activities). The Cronbach’s standardized α was high (> 0.70) for all KCCQ domain/summary scores. In the test–retest analysis among 58 stable patients, all domain/summary scores minimally changed by 0.3–4.2 points with intraclass correlation coefficients of 0.65–0.84, demonstrating moderate to good reproducibility, except for the symptom stability domain. Among 44 patients with improved health states, all domain/summary scores except for the symptom stability and self-efficacy domains substantially improved from baseline with a medium to large effect size of 0.62–0.88.

**Conclusions:**

The Japanese version of the KCCQ was demonstrated to be a valid and reliable tool for the assessment of symptoms and physical function of Japanese patients with chronic heart failure.

## Background

Heart failure (HF) is a clinical syndrome caused by a structural and/or functional cardiac abnormality that is characterized by signs and symptoms such as breathlessness, ankle swelling, and fatigue [1]. HF is a public health concern worldwide with a prevalence of 1–4% in most European countries [2], with the prevalence and incidence increasing progressively with age. In the US, the incidence of HF is reported to be 10 per 1000 after 65 years of age [3]. In Japan, the number of patients with HF is expected to increase rapidly with the aging of the population, and the number of patients with left ventricular dysfunction is estimated to reach 1.3 million by 2030 [4].

Although the prognosis of patients with HF has been improved with advances in treatments [5], it has been reported that the mortality and hospitalization rates still remain high [3, 6, 7]. A Japanese study reported that the rehospitalization rates for HF within 1 year after discharge were 23.7–25.7% [8]. Moreover, HF significantly affects physical function and health state of patients [9–11]. Thus, the goal of treatment is to improve the overall well-being of patients as well as survival, and the use of patient-reported outcomes (PROs) has been gaining momentum in cardiovascular research [12, 13]. PRO measures are reported by patients and are useful to capture the realities of disease burden and treatment impacts. Disease-specific PRO measures may be more useful than generic instruments because they quantify the health state related to a particular disease and are therefore more sensitive to clinical changes [12].

The Kansas City Cardiomyopathy Questionnaire (KCCQ) [14], originally developed in the English language in 2000, is one such disease-specific PRO measure for HF, which assesses symptoms, physical and social limitations, and health-related quality of life (HRQoL). Having been translated into various languages and validated in many country-specific settings [15–19], the KCCQ is now one of the most widely used PRO measures for patients with HF, along with the Minnesota Living with Heart Failure Questionnaire (MLHFQ) [20]. Among the many existing HF-specific PRO measures, the KCCQ and MLHFQ were the only two measures that fit all eight evaluation criteria (e.g., psychometric properties, feasibility, interpretability, and symptom coverage) in a previous systematic review [21]. One beneficial characteristic of the KCCQ is that it provides a summary score specifically focused on patient symptoms and physical limitations (physical function), along with the overall summary score. Symptoms and physical function are the most relevant domains for the clinical assessment of patients with HF, and these domains are also the main concepts of interest in the development of new HF treatments as they are proximal to patient experience of the disease [22].

A linguistically validated Japanese version of the KCCQ is available and used often in clinical trials involving Japanese HF patients [23–25]. However, the psychometric properties of the tool have not yet been evaluated. Therefore, in this study, we evaluated the validity and reliability of the Japanese version of the KCCQ in Japanese patients with chronic HF, with a focus on its domains and summary scores related to symptoms and physical function.

## Methods

### Sample and design of source trials

Data of Japanese patients with chronic HF were drawn from three phase II trials: the SOCRATES-REDUCED [23], SOCRATES-PRESERVED [24], and ARTS-HF Japan [25].

### SOCRATES studies

The SOCRATES-REDUCED and SOCRATES-PRESERVED studies were both multicenter, international, randomized, double-blind, placebo-controlled, dose-finding, phase II trials of vericiguat in patients with chronic HF. Details of the study methods have been previously described [23, 24]. In brief, patients with worsening chronic HF who had either reduced ejection fraction (EF) (EF < 45%, HFrEF) for the SOCRATES-REDUCED or preserved EF (EF ≥45%, HFpEF) for the SOCRATES-PRESERVED were randomized to one of five treatment arms (4 vericiguat and 1 placebo) and received the treatment for 12 weeks.

In the present study, data from the following assessments of patients’ symptoms, functional status, or health state were analyzed: New York Heart Association (NYHA) class [26] recorded at baseline, and the KCCQ and the EuroQol five-dimension, three-level questionnaire (EQ-5D-3L) [27] scores assessed at baseline and at weeks 4, 8, and 12. The present study did not use other clinical data such as biomarkers (e.g., B-type natriuretic peptide [BNP], NT-proBNP), which have low correlation with the patients’ perception of their own health status [28, 29].

### ARTS-HF Japan

ARTS-HF Japan was a randomized, double-blind, active-comparator-controlled, dose-finding phase IIb trial of finerenone in Japanese patients with worsening chronic HF with reduced EF (< 40%) and type 2 diabetes mellitus and/or chronic kidney disease. Patients were randomized to one of six treatment arms (5 finerenone and 1 eplerenone) and received the treatment for 90 days. More detailed study methods including inclusion/exclusion criteria have been described previously [25]. Data from the following assessments were used in the present analysis: NYHA class at baseline and the KCCQ and EQ-5D-3L scores at baseline, days 30 and 90, and 30 days after the last day of treatment (follow-up visit).

### Clinical and health state measures

#### NYHA class

NYHA classification is a system to categorize the extent of physical limitations in patients with HF [26]. Physicians classify patients into one of four classes based on their functional limitations and symptom severity: I (no limitations of physical activity); II (slight limitation); III (marked limitation); and IV (unable to carry on any physical activity without discomfort).

#### KCCQ

The KCCQ is a 23-item (15 questions), self-administered questionnaire quantifying the following clinically relevant domains: physical limitations, symptom frequency, symptom severity, symptom stability, self-efficacy, social limitation, and QoL [14]. The questions refer to the patient’s heart failure symptoms over the past 2 weeks, and each item is scored on a 5- to 7-point Likert scale. A missing value is assigned the average score of the scored items within the domain, and all item scores are summed within each domain. A domain score is transformed to a 0 to 100 scale, with a higher score indicating a better state. Three summary scores are calculated as follows: 1) the total symptom score (TSS)—the average of the symptom frequency and symptom severity domain scores; 2) the clinical summary score (CSS)—the average of the physical limitations domain score and the TSS; and 3) the overall summary score (OSS)—the average of the CSS and the QoL and social limitations domain scores. The symptom stability and self-efficacy domains are not incorporated into any of the KCCQ summary scores [14]. The Japanese version of the KCCQ was translated and linguistically validated by the Mapi Research Institute (Lyon, France).

#### EQ-5D-3L

The EQ-5D-3L is a generic HRQoL measure, consisting of a five-dimension descriptive system and visual analogue scale (VAS) [27]. In the descriptive system, mobility, self-care, usual activities, pain/discomfort, and anxiety/depression are each rated on a 3-point scale (1 = no problems, 2 = some problems, 3 = extreme problems). A patient’s responses to these five dimensions are then converted into a Japanese value set describing the patient’s overall health state, which ranges from − 0.111 to 1.000 (a higher value indicates a better health state) [30]. The EQ-5D VAS records the patient’s health state on a scale of 0 (worst imaginable) to 100 (best imaginable).

### Statistical analyses

Pooled data of Japanese patients with chronic HF from the above-described three trials were analyzed to evaluate the validity and reliability of the Japanese version of the KCCQ. Since symptoms and physical function are more proximal to the patient experience of the disease, our particular focus was on the CSS, a summary scale of symptoms and physical function, and its component domains (i.e., physical limitations, symptom frequency, symptom severity, and TSS). However, every domain and the OSS were also evaluated in this study. Analyses were performed using SAS Release 9.4 (SAS Institute Inc., Cary, NC, USA).

#### Validity

Construct validity was assessed by the known-group analysis, in which we assessed whether the KCCQ scores could differentiate different groups of patients using the NYHA classes to represent groups of patients with different levels of disease severity. The baseline KCCQ scores were summarized for each NYHA class at baseline. To test an increasing or decreasing trend in scores across NYHA classes, the Jonckheere-Terpstra test [31] was performed.

To further evaluate whether the KCCQ scores measured the constructs of interest, correlations between the baseline scores of the KCCQ and a related but different measure, the EQ-5D-3L, were analyzed using the Pearson’s correlation for the EQ-5D VAS and the Spearman rank correlation for the five EQ-5D dimensions. The physical limitations domain score and CSS were both expected to have a moderate correlation with the three EQ-5D dimensions (i.e., mobility, self-care, and usual activities), which are considered to be related to functional domains. The symptom stability domain assesses the change in symptoms over the past 2 weeks, and the self-efficacy domain assesses knowledge or understanding of how to manage their symptoms. As these two domains assess distinctively different concepts from those evaluated by the EQ-5D dimensions, no meaningful correlation was expected between these domains and the EQ-5D-3L.

#### Reliability

To assess whether items designed to measure the same construct actually do so, the internal consistency of each KCCQ domain/summary score, except for the symptom stability domain, which is a single-item domain, was assessed using Cronbach’s standardized α. An α of ≥0.7 is considered to indicate good interrelatedness among the items within the domain or summary score [32].

Test-retest reliability, or reproducibility, was assessed by analyzing whether the scores were stable when the patients’ conditions did not change. The test-retest analysis included patients in a stable condition, which was defined as no change in EQ-5D-3L scores between two timepoints [33]: between week 8 and week 12 for the SOCRATES studies and between the last day of treatment and 30 days after the last treatment for the ARTS-HF Japan study. The concordance of the scores at these two timepoints was evaluated using the intraclass correlation coefficient (ICC) [34]. An ICC of ≥0.7 is considered to indicate good agreement [35], i.e., good reproducibility of the scale.

#### Responsiveness

Responsiveness to patients’ clinical change was evaluated by analyzing whether the KCCQ scores improved when the patients’ health states improved. Patients with improved health states were defined as those with improvement in at least one EQ-5D dimension by ≥1 point without worsening in any EQ-5D dimension [33]. We used the EQ-5D to define those who improved because it was shown to be responsive to clinical changes in patients with HF [36]. Among the patients whose health states were expected to show improvement, changes in the KCCQ scores from baseline to 1 month (more precisely, at week 4 for the SOCRATES studies and at day 30 for the ARTS-HF Japan study) was analyzed by calculating the mean change in scores between the two timepoints and the effect size (mean change in score divided by standard deviation [SD] at baseline). An effect size of 0.2 is interpreted as small, 0.5 as medium, and 0.8 as large [37]. Changes in scores between the two timepoints were also tested using a paired *t*-test with equal variances assumed.

## Results

### Patient characteristics

This study used the pooled data of 141 Japanese patients with chronic HF: 30 patients from SOCRATES-REDUCED; 39 patients from SOCRATES-PRESERVED; and 72 patients from ARTS-HF Japan. Although the SOCRATES-PRESERVED contained more female than the other two trials, no noticeable differences were observed for other baseline data such as NYHA class distribution among the three source trials (Additional file 1).

**Fig. 1:**
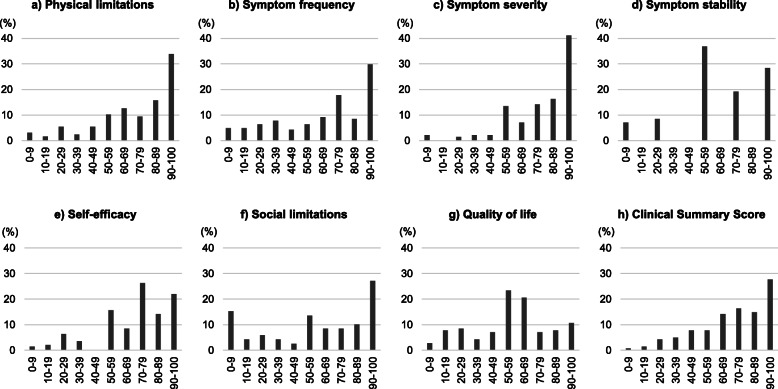
Distribution of the KCCQ domain scores and clinical summary score at baseline (*n* = 141^a^). ^a^*N* = 127 for (**a**) physical limitations domain score due to responses coded as missing data in question 1 (6 items), and *N* = 118 for (**f**) social limitations domain score due to responses coded as missing data in question 15 (4 items). KCCQ, Kansas City Cardiomyopathy Questionnaire

The mean age ± SD of the pooled sample was 73.7 ± 10.9 years, and 71.6% were male (Table 1). Patients with HFrEF accounted for 72.3% of the sample. The majority of patients were classified as NYHA class II (76.6%) at baseline, followed by class III (12.8%), class I (8.5%), and class IV (2.1%). At baseline, all patients responded to all 23 items of the KCCQ (no missing responses). Table 2 summarizes the KCCQ scores at baseline, and Fig. 1 shows the distribution of each domain score and the CSS at baseline. The mean ± SD KCCQ CSS at baseline was 71.6 ± 23.0. As shown in Fig. 1h, the score distribution was negatively skewed (skewness value − 0.75); over 70% of patients had a CSS of ≥60, while 27.7% of patients had a CSS of ≥90.

**Table 1:** Baseline characteristics of the pooled population of Japanese patients with chronic heart failure

	Patients (*n* = 141)
Age (years)	73.7 ± 10. 9
Sex, n (%)	
Male	101 (71.6)
Female	40 (28.4)
Ejection fraction, n (%)	
HFrEF	102 (72.3)
HFpEF	39 (27.7)
NYHA class, n (%)	
I	12 (8.5)
II	108 (76.6)
III	18 (12.8)
IV	3 (2.1)
EQ-5D VAS	64.2 ± 18.0
EQ-5D-3L^a^	0.8 ± 0.2

Data were expressed as mean ± standard deviation or n (%)

^a^The value ranges from −0.111 to 1.000

*HFrEF* heart failure with reduced ejection fraction; *HFpEF* heart failure with preserved ejection fraction; *NYHA* New York Heart Association; *EQ-5D VAS* EuroQol five-dimension visual analogue scale; *EQ-5D-3L* EuroQol five-dimension, three-level questionnaire

**Table 2:** KCCQ scores at baseline

KCCQ	n	Scores (mean ± SD)
Domains		
Physical limitations	127^a^	71.8 ± 25.5
Symptom frequency	141	66.6 ± 29.7
Symptom severity	141	78.1 ± 21.4
Symptom stability	141	63.3 ± 29.8
Self-efficacy	141	70.4 ± 25.0
Social limitations	118^b^	59.1 ± 34.8
Quality of life	141	55.9 ± 24.2
Summary scores		
Total symptom score	141	72.4 ± 24.7
Clinical summary score	141	71.6 ± 23.0
Overall summary score	141	64.6 ± 23.0

^a^Due to responses coded as missing data in question 1 (6 items)

^b^Due to responses coded as missing data in question 15 (4 items)

*KCCQ* Kansas City Cardiomyopathy Questionnaire; *SD* standard deviation

### Validity

**Fig. 2:**
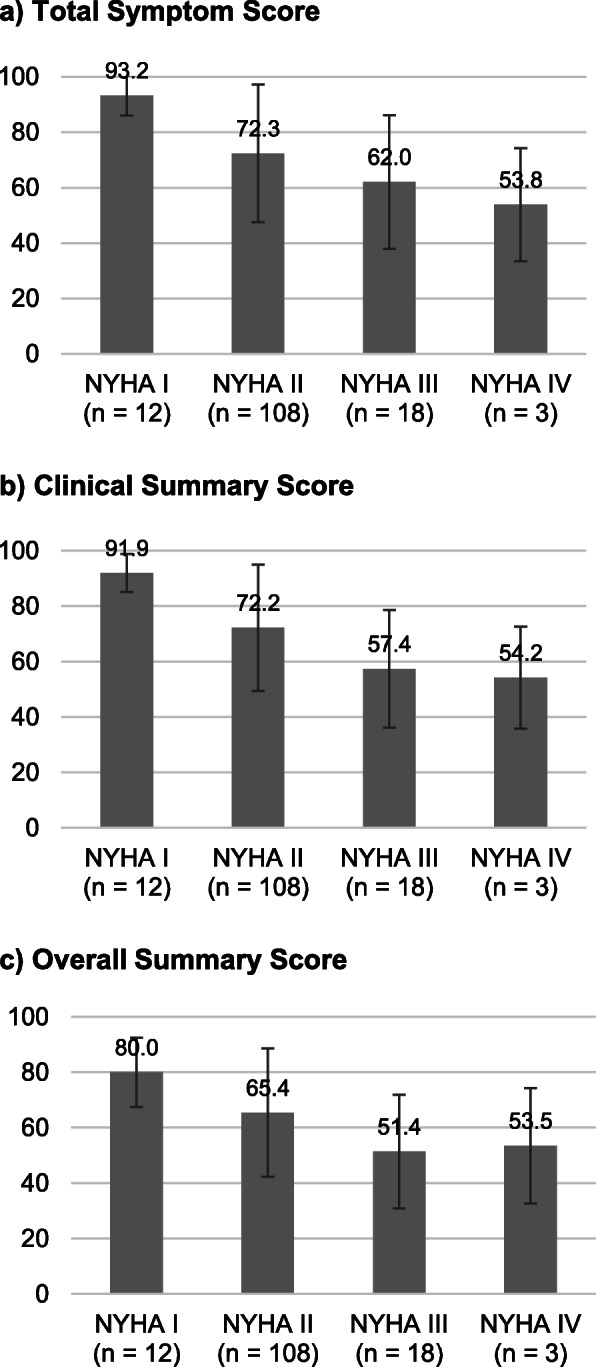
Mean KCCQ summary scores at baseline by NYHA class. KCCQ, Kansas City Cardiomyopathy Questionnaire; NYHA, New York Heart Association

Mean baseline CSSs were lower in patients with higher NYHA classes (91.9 for NYHA class I, 72.2 for class II, 57.4 for class III, and 54.2 for class IV), with a decreasing trend in the scores across NYHA classes (*p* <  0.001, Jonckheere-Terpstra test) (Fig. 2b & Table 3). A decreasing trend was also observed for all three component domains of the CSS and the other summary scores (*p* <  0.001 for all; Table 3, Fig. 2), indicating that symptoms or physical function-related domains and all KCCQ summary scores can differentiate patients with different disease severity. As for other domains, the QoL domain scores were significantly lower in patients with higher NYHA classes (*p* = 0.003), but such a trend was not observed for the symptom stability, self-efficacy, and social limitations domains.

**Table 3:** Known-group analysis: baseline scores of the KCCQ by NYHA class

KCCQ	Scores (mean ± SD)				*P* value^a^
	NYHA I (*n* = 12)	NYHA II (*n* = 108)	NYHA III (*n* = 18)	NYHA IV (*n* = 3)	
Domains					
Physical limitations^b^	90.6 ± 9.3	73.2 ± 25.1	51.4 ± 24.7	64.6 ± 2.9	< 0.001
Symptom frequency	91.3 ± 10.3	66.6 ± 29.9	54.2 ± 27.1	43.8 ± 32.3	< 0.001
Symptom severity	95.1 ± 6.6	78.0 ± 21.2	69.9 ± 24.6	63.9 ± 9.6	< 0.001
Symptom stability	64.6 ± 22.5	64.4 ± 30.0	54.2 ± 33.5	75.0 ± 25.0	0.592
Self-efficacy	77.1 ± 21.9	69.3 ± 25.4	75.7 ± 19.9	50.0 ± 45.1	0.692
Social limitations^c^	59.7 ± 40.8	61.4 ± 34.6	44.8 ± 31.1	54.2 ± 41.2	0.151
Quality of life	73.6 ± 16.2	55.8 ± 24.4	44.4 ± 21.6	55.6 ± 24.1	0.003
Summary scores					
Total symptom score	93.2 ± 7.2	72.3 ± 24.8	62.0 ± 24.1	53.8 ± 20.4	< 0.001
Clinical summary score	91.9 ± 6.8	72.2 ± 22.8	57.4 ± 21.3	54.2 ± 18.4	< 0.001
Overall summary score	80.0 ± 12.5	65.4 ± 23.2	51.4 ± 20.5	53.5 ± 20.8	< 0.001

^a^The trend was tested by the Jonckheere-Terpstra test

^b^Calculated for 127 patients (*n* = 12 for NYHA I, 96 for NYHA II, 17 for NYHA III, and 2 for NYHA IV) due to responses coded as missing data in question 1 (6 items)

^c^Calculated for 118 patients (*n* = 11 for NYHA I, 91 for NYHA II, 14 for NYHA III, and 2 for NYHA IV) due to responses coded as missing data in question 15 (4 items)

*KCCQ* Kansas City Cardiomyopathy Questionnaire; *NYHA* New York Heart Association; *SD* standard deviation

Table 4 summarizes the correlations between the KCCQ and the EQ-5D-3L. The CSS was moderately correlated with the three EQ-5D dimensions (mobility, *ρ* = − 0.54; self-care, *ρ* = − 0.41; and usual activities, *ρ* = − 0.45), as was the physical limitations domain (*ρ* = − 0.46, − 0.40, and − 0.44, respectively). The correlation coefficient with the EQ-5D VAS, a more general measure of health, was low (*r* <  0.3) for all the KCCQ scores except for the physical limitations and QoL domains, and the OSS. As expected, the symptom stability and self-efficacy domains had no correlations with any of the EQ-5D dimensions.

**Table 4:** Correlations between the KCCQ and the EQ-5D-3L

KCCQ	EQ-5D VAS	EQ-5D descriptive system				
		Mobility	Self-care	Usual activities	Pain/discomfort	Anxiety/depression
Domains						
Physical limitations	0.34	−0.46	−0.40	−0.44	−0.27	−0.33
Symptom frequency	0.26	−0.49	−0.35	−0.41	−0.24	−0.29
Symptom severity	0.22	−0.48	−0.31	−0.37	−0.25	−0.27
Symptom stability	0.19	−0.24	−0.09	−0.12	−0.18	−0.09
Self-efficacy	0.13	−0.16	−0.07	−0.15	−0.16	−0.20
Social limitations	0.26	−0.32	−0.19	−0.43	−0.08	−0.28
Quality of life	0.31	−0.46	−0.28	−0.37	−0.23	−0.32
Summary scores						
Total symptom score	0.25	−0.51	−0.34	−0.41	−0.25	−0.29
Clinical summary score	0.29	−0.54	−0.41	−0.45	−0.27	−0.32
Overall summary score	0.32	−0.51	−0.36	−0.47	−0.21	−0.34

Pearson’s correlation coefficients are displayed for the EQ-5D VAS and Spearman rank correlation coefficients for others

*KCCQ* Kansas City Cardiomyopathy Questionnaire; *EQ-5D-3L* EuroQol five-dimension, three-level questionnaire; *EQ-5D VAS* EuroQol five-dimension visual analogue scale

### Reliability

The Cronbach’s standardized α was high for all KCCQ scores (Table 5), indicating good internal consistency for all domain scores (α = 0.74–0.88) and excellent consistency for all summary scores including the CSS (α = 0.90 for all).

**Table 5:** Internal consistency of the domains and summary scores of the KCCQ

KCCQ	Cronbach’s standardized α
Domains	
Physical limitations	0.88
Symptom frequency	0.83
Symptom severity	0.74
Self-efficacy	0.74
Social limitations	0.77
Quality of life	0.75
Summary scores	
Total symptom score	0.90
Clinical summary score	0.90
Overall summary score	0.90

*KCCQ* Kansas City Cardiomyopathy Questionnaire

Test-retest reliability, or reproducibility, was analyzed using data of 58 patients who were considered clinically stable between the two timepoints (i.e., weeks 8 and 12 for the SOCRATES studies, and the last day of treatment and 30 days after the last treatment for the ARTS-HF Japan study). The demographic characteristics of these 58 patients were similar to those of the entire pooled sample (mean age ± SD, 74.1 ± 11.6 years; male, 72.4%) (Additional file 2). As shown in Table 6, scores changed only minimally between the two timepoints (by 0.3–4.2 points on a 100-point scale) for all domain/summary scores. Although moderate reproducibility was demonstrated for the QoL (ICC = 0.65), self-efficacy (0.66), symptom severity (0.68), and physical limitations (0.69) domains, the ICCs were high (> 0.7) for all summary scores including the CSS, indicating high reproducibility for these scales. The only exception was the symptom stability domain, which had a low ICC of 0.19.

**Table 6:** Test-retest analysis: changes in KCCQ scores among clinically stable patients

KCCQ	N	Scores (mean ± SD)			ICC (95% CI)
		Timepoint 1^a^	Timepoint 2^b^	Change in scores	
Domains					
Physical limitations	56^c^	91.0 ± 11.9	93.1 ± 11.1	2.3 ± 8.9	0.69 (0.52–0.81)
Symptom frequency	58	91.2 ± 14.9	90.7 ± 13.8	−0.5 ± 9.6	0.78 (0.65–0.86)
Symptom severity	58	94.5 ± 9.6	95.5 ± 7.4	1.0 ± 6.8	0.68 (0.51–0.80)
Symptom stability	58	60.3 ± 19.9	59.9 ± 19.3	−0.4 ± 25.0	0.19 (− 0.08–0.43)
Self-efficacy	58	82.8 ± 19.6	80.6 ± 18.8	−2.2 ± 15.9	0.66 (0.48–0.78)
Social limitations	52^d^	93.2 ± 13.9	92.9 ± 15.6	0.5 ± 10.1	0.76 (0.61–0.86)
Quality of life	58	81.0 ± 13.5	85.2 ± 14.0	4.2 ± 11.1	0.65 (0.45–0.78)
Summary scores					
Total symptom score	58	92.9 ± 11.9	93.1 ± 10.0	0.3 ± 7.6	0.77 (0.63–0.85)
Clinical summary score	58	92.0 ± 10.1	93.2 ± 9.0	1.1 ± 5.9	0.80 (0.69–0.88)
Overall summary score	58	89.4 ± 9.9	91.1 ± 10.4	1.7 ± 5.5	0.84 (0.74–0.91)

Test-retest analysis was conducted using data of 58 patients who were considered clinically stable, which was defined as no change in EQ-5D-3L scores, between the two timepoints

^a^At week 8 for the SOCRATES studies and at last day of treatment for the ARTS-HF Japan study

^b^At week 12 for the SOCRATES studies and at 30 days after the last treatment for the ARTS-HF Japan study

^c^Data of 56 patients were analyzed due to responses coded as missing data in question 1 (6 items)

^d^Data of 52 patients were analyzed due to responses coded as missing data in question 15 (4 items)

*KCCQ* Kansas City Cardiomyopathy Questionnaire; *ICC* intraclass correlation coefficient; *CI* confidence interval; *SD* standard deviation

### Responsiveness

Responsiveness of the KCCQ was analyzed using the data of 44 patients who showed improvement in their health state. Changes in the KCCQ scores among these patients are summarized in Table 7. For the three component domains of the CSS, scores significantly increased after 1 month of treatment (*p* <  0.001 for all, paired *t*-test), with the greatest increase in the symptom frequency domain score by 26.9 points. The social limitations and QoL domain scores also substantially increased by more than 20 points, but the symptom stability and self-efficacy domain scores did not largely change with a small effect size of < 0.4. All three summary scores including the CSS substantially increased by more than 20 points with a large effect size of > 0.80, demonstrating the substantial responsiveness of the KCCQ to changes in patients’ clinical status.

**Table 7:** One-month change in KCCQ scores among patients with improved health states

KCCQ	N	Scores (mean ± SD)			Effect size	*P* value^b^
		Baseline	After one month of treatment^a^	Change in scores		
Domains						
Physical limitations	35^c^	66.4 ± 27.6	83.6 ± 18.2	17.2 ± 24.2	0.62	< 0.001
Symptom frequency	44	55.1 ± 31.6	82.0 ± 16.9	26.9 ± 30.2	0.85	< 0.001
Symptom severity	44	69.3 ± 25.5	87.5 ± 14.5	18.2 ± 24.4	0.71	< 0.001
Symptom stability	44	61.9 ± 33.4	69.9 ± 21.3	8.0 ± 42.4	0.24	0.220
Self-efficacy	44	66.5 ± 21.9	74.1 ± 23.9	7.7 ± 16.7	0.35	0.004
Social limitations	30^d^	60.8 ± 31.4	81.2 ± 20.9	20.5 ± 28.4	0.65	< 0.001
Quality of life	44	47.3 ± 26.0	68.0 ± 19.3	20.6 ± 26.4	0.79	< 0.001
Summary scores						
Total symptom score	44	62.2 ± 27.3	84.8 ± 15.0	22.5 ± 25.7	0.82	< 0.001
Clinical summary score	44	62.0 ± 25.7	83.7 ± 13.9	21.7 ± 21.3	0.85	< 0.001
Overall summary score	44	56.4 ± 25.3	78.5 ± 15.3	22.2 ± 20.7	0.88	< 0.001

Responsiveness was analyzed using data of 44 patients who showed improvement in their health state, which was defined as improvement in at least one EQ-5D dimension by ≥1 point without worsening in any EQ-5D dimension

^a^For the assessment of this timepoint, the KCCQ scores at week 4 for the SOCRATES studies and at day 30 for the ARTS-HF Japan study were used

^b^Differences in scores between baseline and the second assessment were tested using a paired *t*-test with equal variances assumed

^c^Data of 35 patients were analyzed due to responses coded as missing data in question 1 (6 items)

^d^Data of 30 patients were analyzed due to responses coded as missing data in question 15 (4 items)

Effect sizes: 0.2 = small; 0.5 = medium; 0.8 = large

*KCCQ* Kansas City Cardiomyopathy Questionnaire; *SD* standard deviation

## Discussion

The use of a valid PRO measure is essential for the adequate assessment of patients’ health states. In this study, to assess the psychometric properties of the Japanese version of the KCCQ, we evaluated the validity and reliability of the tool with a focus on the CSS and its component domains, which are considered most relevant for the clinical assessment of patients’ symptoms and physical functioning. The results of this study demonstrated that the Japanese version of the KCCQ had construct validity, good internal consistency, and high reproducibility and responsiveness when used in Japanese patients with chronic HF.

The known-group analysis showed that the three symptoms or physical function-related domain scores (i.e., physical limitations, symptom frequency, and symptom severity) and KCCQ summary scores were all associated with NYHA class, indicating that these scores accurately differentiated patients with differing disease severity. However, the social limitation domain score did not show a decreasing trend with the NYHA class. This result may be due to the disproportionate distribution of patients across NYHA classes (i.e., few patients were in higher NYHA classes III and IV) in this pooled sample. In addition, there was a response option that was coded as missing, which further contributed to the small number of patients with analyzable data in this domain. Although the known-group validity of this domain remains to be confirmed, a moderate correlation of this domain with the EQ-5D usual activity (*ρ* = − 0.43) partially supports its construct validity. The construct validity of the tool for the assessment of patients’ symptoms and physical functioning was further supported by moderate correlations of the CSS and physical limitations domain with the three EQ-5D dimensions that are related to functional domains. Considering that the EQ-5D-3L is a generic measure and the KCCQ is a HF-specific measure, their scores do not represent an exactly comparable assessment of domains, leading to understandably moderate rather than high correlation.

For reliability, all KCCQ domain/summary scores showed good internal consistency, as demonstrated by a high Cronbach’s α (> 0.7), which indicates that the items constituting the domain or summary scale can be considered to measure the same construct. In particular, the CSS had excellent internal consistency with an α of 0.90, which was almost equivalent to that of its original KCCQ counterpart (α = 0.93 [14]). In the test-retest analysis using clinically stable patients, minimal changes in scores between the two assessments with ICCs of 0.69–0.78 demonstrated the moderate to high reproducibility of the three component domains of the CSS. The CSS and the other two summary scores also had high ICCs of 0.77–0.84, showing good reproducibility of these scales. The mean changes in scores between the two assessments were minimal (by 0.4–4.2 points on a 100-point scale) for other domains as well; however, the ICC of the symptom stability domain was exceptionally low (ICC = 0.19). This was probably because this is a single-item domain, and thus even a one-point change on a 5-point scale in a patient’s response was converted into a substantial score change on a scale of 0 to 100 for the domain score.

One advantage of the KCCQ over the MLHFQ is that the KCCQ is more sensitive to clinical change [14]. Although a comparison with existing tools could not be performed in this study due to secondary use of trial data, our analyses showed that the Japanese version of the KCCQ was highly responsive to patients’ clinical change. All domain scores significantly increased by 17.2–26.9 points after 1 month of treatment in patients with improved health states, except for the symptom stability and self-efficacy domains. In particular, the symptom frequency domain and all summary scores, including the CSS, showed especially high responsiveness with a large effect size (> 0.80). However, the responsiveness of the symptom stability and self-efficacy domains, neither of which are incorporated into any of the KCCQ summary scores, could not be confirmed in this analysis. As they are conceptually different from other domains, their responsiveness may need to be evaluated in a more appropriate method.

In the development study of the original KCCQ, the baseline CSS was significantly lower in patients who subsequently died or required rehospitalization than in event-free survivors (35.1 vs. 55.3, *p* <  0.001), suggesting the prognostic value of the tool [14]. Unfortunately, we were unable to assess the prognostic value of the Japanese version of the KCCQ in this study owing to certain methodological limitations, such as a small number of patients with few numbers of prognostic events, which may be due to a short observation period and a disproportionately large proportion of patients with less severe symptoms (85.1% were classed as NYHA class I–II at baseline), as well as confounding by treatment effects (e.g., patients received different treatments according to their treatment group). The prognostic value of the Japanese version of the KCCQ would be worthy of further investigation.

PRO measures have been historically underused as metrics in clinical studies [12]. However, in light of the increased focus on improving the overall well-being of patients, they are encouraged to be used as endpoints in cardiovascular studies [13], and selected KCCQ domains are increasingly being used as such in heart failure trials. The KCCQ not only assesses all three principal components of patients’ health states, i.e., symptoms, functional status, and HRQoL, but can also be an independent predictor of poor prognosis [38] and future healthcare costs [39]. In addition, because the KCCQ is available in many languages, its use as a metric in clinical studies would enable international comparison of the health states of patients with HF. Furthermore, the KCCQ may also help to enhance patient care by directly informing clinicians of the patients’ disease burden and treatment impacts when used in clinical setting. Continued exploration of the usefulness of the KCCQ in clinical practice is warranted in future studies.

This study has several limitations. First, because this study involved the secondary use of three trials’ data and analyzed a pooled sample, the generalizability of the results of this study may be limited by the inclusion/exclusion criteria of the source trials. For example, as the majority of patients (76.6%) were classed as NYHA class II at baseline, our results may not be applicable to patients with more severe symptoms. Second, construct validity was assessed using only NYHA class and the EQ-5D-3L because of the limited measures available in the secondary use of trial data. For the symptoms and physical function-related scales of the KCCQ, assessment of correlations with measures with more similar constructs (e.g., MLHFQ) and measures that assess related functional domains (e.g., 6-min walk test) would have been useful. Moreover, construct validity of other domains, especially the self-efficacy and social limitations domains, require further evaluation using a more related, appropriate reference measures for each domain. Third, the reliability and validity of the single-item, symptom stability domain could not be confirmed in the present analysis. This item is inherently different from the other KCCQ items because it asks the patient to rate the degree of change in their symptoms over the past 2 weeks. Therefore, it was not expected to perform similarly to the other items and domain scores that do not require a comparison of current and previous experiences. Further assessment of this domain is warranted. However, the present analysis confirmed the reliability and validity of the CSS, the most relevant KCCQ summary score for clinical assessment. Thus, we believe that our results would provide valuable information for users of the Japanese version of the KCCQ. Lastly, although we defined patients’ symptom stability and changes in clinical status using the EQ-5D-3L, which has been reported to be responsive to clinical changes in patients with HF [36], they may not have been adequately captured by the EQ-5D-3L. The EQ-5D-3L may be responsive to only relatively large changes, thereby limiting the analysis sample for the assessment of responsiveness, which may have contributed somewhat to the better responsiveness of the KCCQ. Likewise, although we observed robust stability estimated in the test–retest analysis, the analysis may have included some patients who had clinical changes.

## Conclusions

This study showed that the Japanese version of the KCCQ, especially its scales related to symptoms and physical functioning, is a valid and reliable measure, with construct validity, good internal consistency, and high reproducibility and responsiveness. Further evaluation of the psychometric properties of some domains as well as its prognostic value is warranted in Japanese patients with HF.

## Supplementary information

**Additional file 1.** Baseline characteristics and KCCQ scores of patients in each source trial. Patient characteristics and baseline KCCQ scores are summarized by each trial.

**Additional file 2 **Baseline characteristics of patients who were clinically stable between the two timepoints. Patient characteristics (*n* = 58) are summarily analyzed for test-retest reliability.

## Data Availability

The datasets used and analyzed during the current study are available from the corresponding author upon reasonable request.

## Abbreviations

HF: Heart failure
EF: Ejection fraction
HFpEF: Heart failure with preserved ejection fraction
HFrEF: Heart failure with reduced ejection fraction
PRO: Patient-reported outcome
KCCQ: The Kansas City Cardiomyopathy Questionnaire
HRQoL: Health-related quality of life
NYHA: New York Heart Association
EQ-5D-3L: EuroQol, five-dimension, three-level
TSS: Total symptom score
CSS: Clinical summary score
VAS: Visual analogue scale
ICC: Intraclass correlation coefficient
SD: Standard deviation
