# Supplementary material for: Psychometric properties of the Japanese version of the Kansas City Cardiomyopathy Questionnaire in Japanese patients with chronic heart failure
Source: Health Qual Life Outcomes. 2020 Jul 17;18:236. doi: 10.1186/s12955-020-01483-0 (PMC7368765; doi:10.1186/s12955-020-01483-0)
Supplement: Supplementary file 1 — Additional file 1. Baseline characteristics and KCCQ scores of patients in each source trial. Patient characteristics and baseline KCCQ scores are summarized by each trial. [file 12955_2020_1483_MOESM1_ESM.docx]

**Additional file 1. Baseline characteristics and KCCQ scores of patients in each source trial**

|  | Pooled population  (n = 141) |  | Patients with HFrEF | | Patients with HFpEF |
| --- | --- | --- | --- | --- | --- |
|  |  |  | SOCRATES-REDUCED  (n = 30) | ARTS-JAPAN  (n = 72) | SOCRATES-PRESERVED  (n = 39) |
| Age (years) | 73.7 ± 10.9 |  | 69.9 ± 14.1 | 73.1 ± 9.3 | 77.7 ± 9.9 |
| Sex, n (%) |  |  |  |  |  |
| Male | 101 (71.6) |  | 26 (86.7) | 53 (73.6) | 22 (56.4) |
| Female | 40 (28.4) |  | 4 (13.3) | 19 (26.4) | 17 (43.6) |
| Ejection fraction, n (%) |  |  |  |  |  |
| HFrEF | 102 (72.3) |  | 30 (100.0) | 72 (100.0) | 0 (0.0) |
| HFpEF | 39 (27.7) |  | 0 (0.0) | 0 (0.0) | 39 (100.0) |
| NYHA class, n (%) |  |  |  |  |  |
| I | 12 (8.5) |  | 4 (13.3) | 0 (0.0) | 8 (20.5) |
| II | 108 (76.6) |  | 23 (76.7) | 57 (79.2) | 28 (71.8) |
| III | 18 (12.8) |  | 3 (10.0) | 12 (16.7) | 3 (7.7) |
| IV | 3 (2.1) |  | 0 (0.0) | 3 (4.2) | 0 (0.0) |
| EQ-5D VAS | 64.2 ± 18.0 |  | 70.3 ± 13.3 | 62.5 ± 19.7 | 62.8 ± 17.3 |
| EQ-5D-3L | 0.8 ± 0.2 |  | 0.9 ± 0.2 | 0.7 ± 0.2 | 0.8 ± 0.2 |
| KCCQ scores |  |  |  |  |  |
| *Domains* |  |  |  |  |  |
| Physical limitations | n = 127, 71.8 ± 25.5 |  | 82.5 ± 18.3 | n = 65, 64.5 ± 27.3 | n = 32, 76.5 ± 23.6 |
| Symptom frequency | 66.6 ± 30.0 |  | 79.7 ± 23.6 | 58.0 ± 30.2 | 72.4 ± 28.3 |
| Symptom severity | 78.1 ± 21.4 |  | 86.4 ± 13.8 | 72.6 ± 22.4 | 82.1 ± 21.9 |
| Symptom stability | 63.3 ± 29.8 |  | 70.0 ± 28.9 | 56.3 ± 31.6 | 71.2 ± 24.0 |
| Self-efficacy | 70.4 ± 25.0 |  | 73.3 ± 23.6 | 69.6 ± 25.6 | 69.6 ± 25.5 |
| Social limitations | n = 118, 59,1 ± 34.8 |  | n = 25, 71.5 ± 31.0 | n = 64, 57.9 ± 35.9 | n = 29, 51.1 ± 33.9 |
| Quality of life | 55.9 ± 24.2 |  | 62.8 ± 19.9 | 50.3 ± 26.2 | 60.7 ± 21.3 |
| *Summary scores* |  |  |  |  |  |
| Total symptom score | 72.4 ± 24.7 |  | 83.1 ± 18.2 | 65.3 ± 25.1 | 77.2 ± 24.6 |
| Clinical summary score | 71.6 ± 23.0 |  | 82.8 ± 15.4 | 64.5 ± 23.7 | 76.1 ± 22.4 |
| Overall summary score^a^ | 64.6 ± 23.0 |  | 74.7 ± 16.4 | 59.3 ± 25.0 | 66.5 ± 20.8 |

Data were expressed as mean ± standard deviation or n (%).

^a^The average of all domain scores except for symptom stability and self-efficacy.

KCCQ, Kansas City Cardiomyopathy Questionnaire; HFrEF, heart failure with reduced ejection fraction; HFpEF, heart failure with preserved ejection fraction; NYHA, New York Heart Association; EQ-5D VAS, EuroQol five-dimension visual analogue scale; EQ-5D-3L, EuroQol five-dimension, three-level questionnaire.
