# Supplementary material for: Psychometric properties of the Japanese version of the Kansas City Cardiomyopathy Questionnaire in Japanese patients with chronic heart failure
Source: Health Qual Life Outcomes. 2020 Jul 17;18:236. doi: 10.1186/s12955-020-01483-0 (PMC7368765; doi:10.1186/s12955-020-01483-0)
Supplement: Supplementary file 2 — Additional file 2 Baseline characteristics of patients who were clinically stable between the two timepoints. Patient characteristics (n = 58) are summarily analyzed for test-retest reliability. [file 12955_2020_1483_MOESM2_ESM.docx]

**Additional file 2. Baseline characteristics of patients who were clinically stable between the two timepoints**

|  | Patients  (n = 58) |
| --- | --- |
|  |  |
| Age (years) | 74.1 ± 11.6 |
| Sex, n (%) |  |
| Male | 42 (72.4) |
| Female | 16 (27.6) |
| Ejection fraction, n (%) |  |
| HFrEF | 39 (67.2) |
| HFpEF | 19 (32.8) |
| NYHA class, n (%) |  |
| I | 8 (13.8) |
| II | 44 (75.9) |
| III | 6 (10.3) |
| IV | 0 (0.0) |
| KCCQ summary scores |  |
| Total symptom score | 79.5 ± 21.6 |
| Clinical summary score | 80.4 ± 19.8 |
| Overall summary score^a^ | 74.5 ± 18.7 |
| EQ-5D VAS | 68.8 ± 17.6 |
| EQ-5D-3L^b^ | 0.8 ± 0.2 |

Data were expressed as mean ± standard deviation or n (%).

^a^The average of all domain scores except for symptom stability and self-efficacy.

^b^The value ranges from −0.111 to 1.000.

HFrEF, heart failure with reduced ejection fraction; HFpEF, heart failure with preserved ejection fraction; NYHA, New York Heart Association; KCCQ, Kansas City Cardiomyopathy Questionnaire; EQ-5D VAS, EuroQol five-dimension visual analogue scale; EQ-5D-3L, EuroQol five-dimension, three-level questionnaire.
